# Endobronchial Mucosa Invasion Predicts Survival in Patients with Small Cell Lung Cancer

**DOI:** 10.1371/journal.pone.0047613

**Published:** 2012-10-04

**Authors:** Pai-Chien Chou, Shu-Min Lin, Chun-Yu Lo, Hao-Cheng Chen, Chih-Wei Wang, Chun-Liang Chou, Chih-Teng Yu, Horng-Chyuan Lin, Chun-Hua Wang, Han-Pin Kuo

**Affiliations:** 1 Department of Thoracic Medicine, Chang Gung Memorial Hospital, Chang Gung University, School of Medicine, Taipei, Taiwan; 2 Department of Pathology, Chang Gung Memorial Hospital, Chang Gung University, School of Medicine, Taipei, Taiwan; National Taiwan University Hospital, Taiwan

## Abstract

**Background:**

Current staging system for small cell lung cancer (SCLC) categorizes patients into limited- or extensive-stage disease groups according to anatomical localizations. Even so, a wide-range of survival times has been observed among patients in the same staging system. This study aimed to identify whether endobronchial mucosa invasion is an independent predictor for poor survival in patients with SCLC, and to compare the survival time between patients with and without endobronchial mucosa invasion.

**Methods:**

We studied 432 consecutive patients with SCLC based on histological examination of biopsy specimens or on fine-needle aspiration cytology, and received computed tomography and bone scan for staging. All the enrolled patients were assessed for endobronchial mucosa invasion by bronchoscopic and histological examination. Survival days were compared between patients with or without endobronchial mucosa invasion and the predictors of decreased survival days were investigated.

**Results:**

84% (364/432) of SCLC patients had endobronchial mucosal invasion by cancer cells at initial diagnosis. Endobronchial mucosal involvement (Hazard ratio [HR], 2.01; 95% Confidence Interval [CI], 1.30–3.10), age (HR, 1.04; 95% CI, 1.03–1.06), and extensive stage (HR, 1.39; 95% CI, 1.06–1.84) were independent contributing factors for shorter survival time, while received chemotherapy (HR, 0.32; 95% CI, 0.25–0.42) was an independent contributing factor better outcome. The survival days of SCLC patients with endobronchial involvement were markedly decreased compared with patients without (median 145 vs. 290, p<0.0001). Among SCLC patients of either limited (median 180 vs. 460, p<0.0001) or extensive (median 125 vs. 207, p<0.0001) stages, the median survival duration for patients with endobronchial mucosal invasion was shorter than those with intact endobronchial mucosa, respectively.

**Conclusion:**

Endobronchial mucosal involvement is an independent prognostic factor for SCLC patients and associated with decreased survival days.

## Introduction

Small cell lung cancer (SCLC), accounting for 20% of lung tumors, is relative sensitive to cytotoxic chemotherapy and radiation therapy [1]. SCLC which arises from or is related to the pulmonary neuroendocrine cell possesses distinctive cellular and molecular biologic characteristics compared with the non-small cell lung cancer [2], [3]. Despite a high response rate to chemotherapy and radiotherapy, SCLC remains associated with a poor long term survival due to its rapid relapse [4].

SCLC patients are staged according to a two-stage system, which was developed by the Veterans Administration Lung Cancer Study Group [5], as having limited stage disease or extensive-stage disease. Clinical stage at initial presentation is one of the most powerful prognostic factors identified in most studies [6], [7]. 60 to 65% of patients have extensive disease at diagnosis with median survival of approximately 9 months, while about 18 months of median survival was reported in patients with limited-stage disease [5]. Due to other independent prognostic factors, there remains heterogeneity among studies [8], [9]. The identification of risk factors could explain the variability observed in survival and be useful in stratification for subgroup for new therapeutic strategies.

SCLC often occurs initially as a centrally located endobronchial tumor [10], a characteristics which made this cancer ideal for bronchoscopic examination. Indeed, the initial histological diagnosis of SCLC is often rendered via bronchoscopic examination [10]. Evidence suggests that abnormal findings in bronchoscopic examination is associated with early relapse or SCLC [11]. In addition, SCLC patients with endobronchial mucosa invasion by tumor are less sensitive to chemotherapy when compared with those without endobronchial mucosa invasion [11]. However, the association between endobronchial mucosa invasion and survival outcome in patients with SCLC has not been well addressed.

This study is designed to identify whether endobronchial mucosa invasion is an independent predictor for poor survival in patients with SCLC, and to compare the survival time between patients with and without endobronchial mucosa invasion in the whole cohort and in subgroups stratified as limited and extensive stages.

## Patients and Methods

### Study Subjects

Medical records from Jan. 1998 through Dec. 2005 were reviewed for all of the patients diagnosed with small cell lung cancer (SCLC) at the Chang Gung Memorial Hospital, Taiwan, a tertiary-care transfer center. The diagnosis was based on histological examination of biopsy specimens or on fine-needle aspiration cytology. There were 765 patients with SCLC during this period of time. All patients had been explained about the indications of the bronchoscopy, which is one part of the diagnostic processes. Among them, 432 patients received bronchoscopic examination for tissue proof and staging of SCLC, and were enrolled in the analysis. Patients without bronchoscopic examination, or patients with previous or co-existent malignancies other than SCLC were excluded from analysis. No additional bronchoscopic examination was performed for this study. The institutional review board of Chang Gung Memorial Hospital approved this study and waived the requirement for informed consent. An IRB certificate of approval of the study in English version has been attached together with the representative patient’s signed permit for bronchoscopic examination in English and Chinese Version in the supporting files.

### Staging and Diagnostic Procedures

All patients had a complete history, physical examination, evaluation of performance status and investigations to define the extent of their disease, which included routine hematology and biochemistry, chest X-ray, bronchoscopy, computed tomography (CT) scan of the thorax, including the upper part of abdomen or ultrasound examination of the abdomen, and radionuclide bone scan. Routine biochemistry included aspartate aminotransferase (AST), alanine aminotransferase (ALT), alkaline phosphatase (AlkP), lactate dehydrogenase (LDH) and measurement of serum electrolytes (sodium, potassium, and chloride), serum creatinine and blood urea nitrogen. A brain CT scan was not mandatory unless neurological symptoms were present. Patients were classified into limited-stage or extensive-stage disease according to Veterans’ Administration Lung Cancer Study Group [5]. Limited disease (LD) was defined as that confined to one thorax, including the bilateral mediastinal and supraclavicular nodes; any involvement beyond these confines was defined as extensive disease (ED). Patients with pleural effusion were included in the ED category. The Eastern Cooperative Oncology Group (ECOG) performance status [12] and location of tumor on initial diagnosis of SCLC were recorded. Extra-pulmonary involvement was defined as SCLC with distant metastasis other than intra-thoracic lymph node metastasis.

### Treatment

The study also recorded administrated therapies in all the patients, including radiotherapy, chemotherapy, concurrent chemo-radiotherapy (CCRT), and best supportive care. Chemotherapy denoted etoposide (60 mg/m^2^) given on day 1 and cisplatin (60 mg/m^2^) given on day 1–3 on a monthly basis for 4–6 cycles as the first line chemotherapy. In patients who are unresponsive to the first line chemotherapy or intolerant of toxicity, topotecan (1.2 mg/m^2^) given on day 1–5 on a monthly basis will be prescribed instead. In this study, 161 patients (51.2%) received less than 3 cycles of chemotherapy, 99 (31.5%) received 3 to 5 cycles of chemotherapy, and 54 (17.2%) received 6 cycles of chemotherapy. There were 30 patients receiving 2^nd^ line topotecan chemotherapy. CCRT included administration of thoracic radiotherapy concurrently with etoposide and cisplatin within the first 6 weeks of chemotherapy.

### Bronchoscopic Study

**Figure 1:**
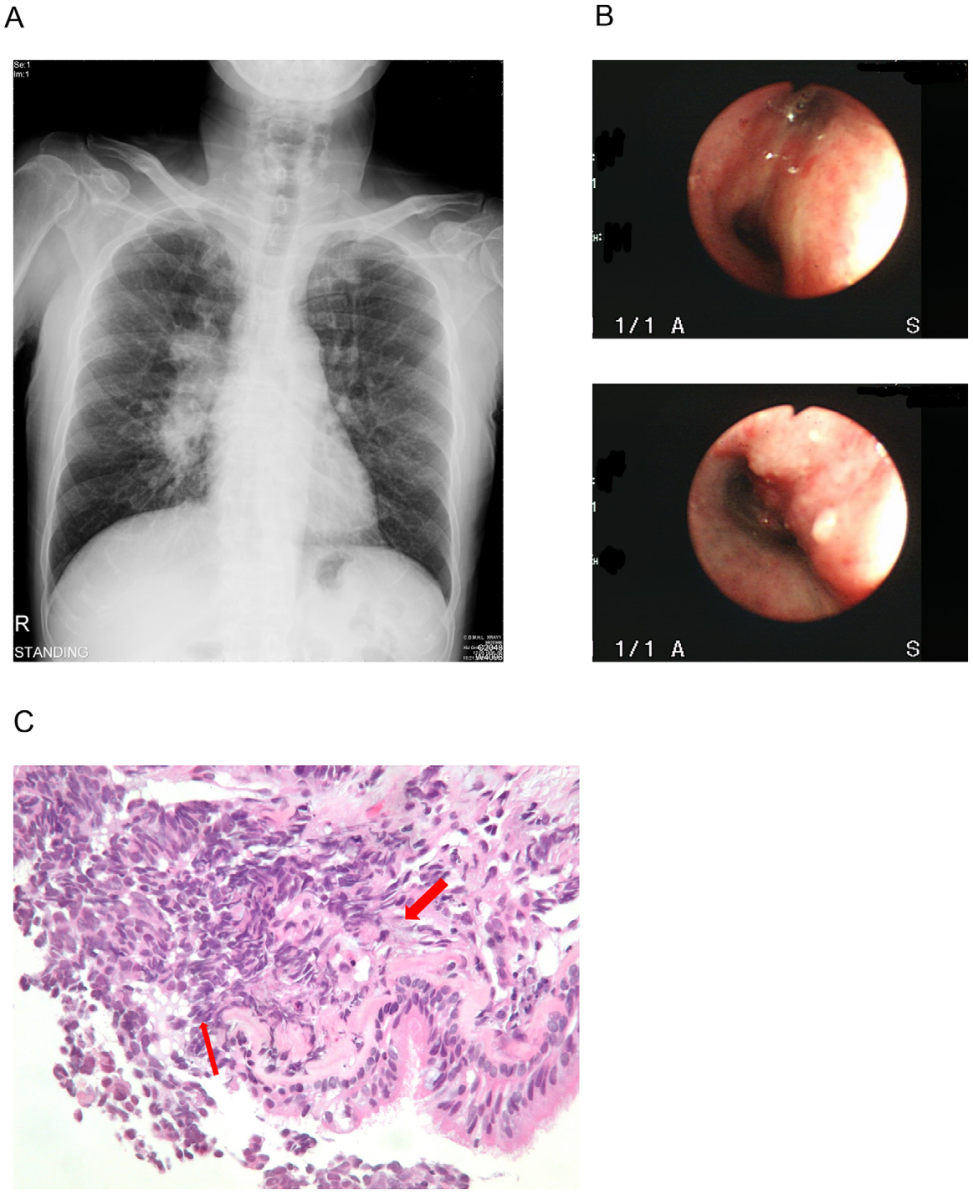
Bronchoscopy identifies mucosal changes and structure abnormality in SCLCs. (A) Initial CXR showed right hilar lesions. (B) Bronchoscopy revealed narrowed bronchus due to external compression (upper figure), and mucosal invasion (lower figure). (C) In histologic examination of the biopsied tissue, endobronchial mucosal invasion (EMI) has been identified with tumor (thin arrow) extends above lamina propria (thick arrow) under high power field examination (400X).

The probable location of the lesion was determined initially by conventional posterior anterior chest radiography with or without chest CT. A flexible fiberoptic bronchoscope (BF-P240 or BF-40, Olympus, Tokyo, Japan) was then inserted via nostril after local spray of 2% xylocaine for anesthesia. The heart rate and oxygen saturation of each patient was monitored by a pulse oximeter during the procedure. In all patients, visual inspection of the larynx, vocal cords, trachea, carina, and all segmental orifices of both lungs was performed. Normal saline solution was instilled through the bronchoscope, with aspiration of washings for cytologic examination. Bronchial biopsies were done with forceps, and a minimum of three biopsies were taken. Bronchial biopsies were performed on suspicious lesions including endobronchial nodules and narrowing or obliteration of the bronchial lumen by swollen mucosa. Mucosal involvement was confirmed by histological examination (Figure 1). The endobronchial mucosal invasion was defined as tumor extends above the lamina propria of the mucosae. Each patient received at least 3 pieces of samples (4.5 mean, range 3 to 6) from the involved site. All samples were embedded in paraffin, and cut at the same thickening at fixed distance into 4 slides. Patient will be classified if any of the slides presented with tumor extends above the lamina propria.

### Statistical Methods

Descriptive statistics were employed to examine the demographic characteristics of the study population. Survival time was expressed as median and Inter-quartile range (IQR). The variables between two groups were compared using the Student t test for continuous variables and the chi-square or the Fisher exact test for categorical variables. Univariate analyses were primarily used for variable selection, based on a p-value under 0.1. The selected variables were entered into a multivariable stepwise regression analysis that identified the net effects of each individual factor. Odds ratios (OR) and their 95% confidence intervals (CI) were used to assess independent contribution of significant factors. The selected variables were further analyzed with the Cox proportional hazard model to adjust for potential confounding effects between each other factor. Hazard ratio (HR) with 95% confidence interval (CI) was used to report the result. The survival curves were estimated by the method of Kaplan and Meier, and the curves were compared according to one factor by the log rank test. Analyses were performed using SPSS software version 12.0 (Chicago, IL, USA).

## Results

### Patient Characteristics

Table 1 lists the patient characteristics, including sex, age, smoking status, performance status, clinical staging, chemotherapy received and tumor location. There were 364 (84%) patients found to have endobronchial mucosal invasion by cancer cells on bronchoscopic examination. The characteristics of patients with or without endobronchial mucosal invasion are similar (Table 1), though patients with endobronchial mucosa invasion tended to have less courses of chemotherapy due to poor response to chemotherapy and more rapid disease progression.

**Table 1:** Patient characteristics of mucosal involvement in SCLC^a^ patients.

Characteristic	Mucosa involvedN = 364	Mucosa uninvolvedN = 68	*P*-value
Age, yr, mean±SD^b^	66.7±10.1	65.2±11.4	.300
Female sex, No. (%)	25(6.9%)	8(11.8%)	.209
ECOG^c^ scale ≤ 2	274(75.3%)	55(80.9%)	.356
Extensive stage	208(57.1%)	37(54.4%)	.691
Chemotherapy ≥4	67 (32.2%)	18 (48.6%)	.061
Extrapulmonary involvement	152(41.8%)	25(36.8%)	.503
Tumor Location, no (%)			
Mediastinum	121(33.2%)	29(42.6%)	.165
Left lung	125(34.3%)	18(26.5%)	.261
Right lung	118(32.4%)	21(30.9%)	.888

a SCLC: Small cell lung cancer.

b SD: Standard deviation.

c ECOG: The Eastern Cooperative Oncology Group performance status.

### The Duration of Survival

**Figure 2:**
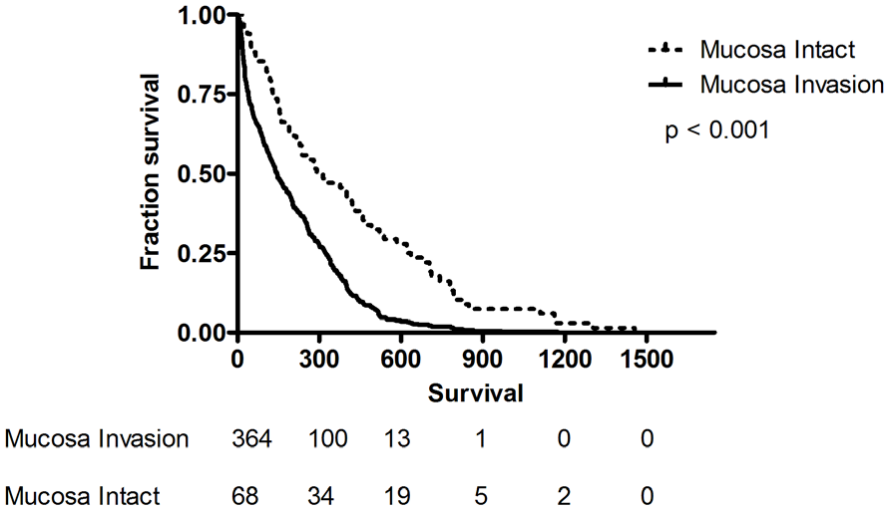
Survival proportion for patients with or without endobronchial mucosa invasion were traced with the Kaplan-Meier method (log rank test, <0.0001). The dashed line represents patients with intact mucosa; the continuous line represents patients with mucosa invasion.

**Figure 3:**
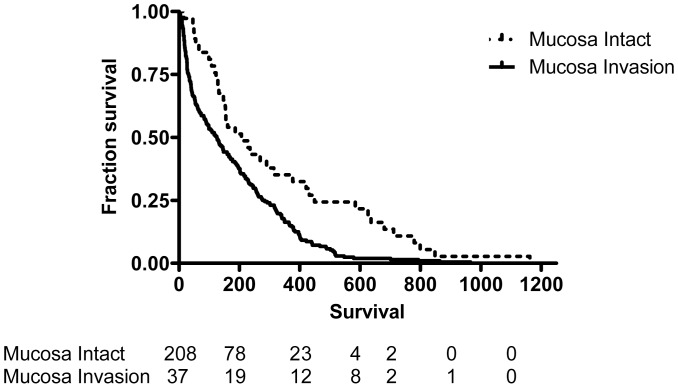
Survival proportion for extensive stage patients with or without endobronchial mucosa invasion were traced with the Kaplan-Meier method (log rank test, p = 0.0005). The dashed line represents patients with intact mucosa; the continuous line represents patients with mucosa invasion.

**Figure 4:**
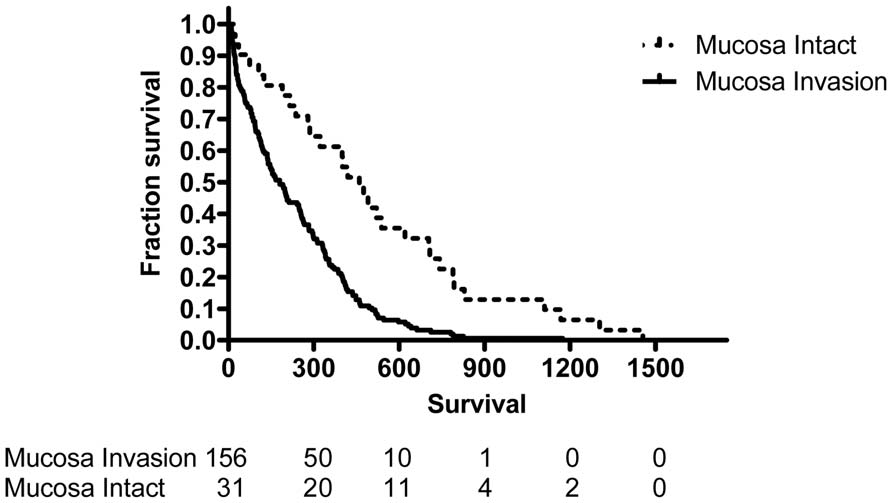
Survival proportion for limited stage patients with or without endobronchial mucosa invasion were traced with the Kaplan-Meier method (log rank test, p<0.0001). The dashed line represents patients with intact mucosa; the continuous line represents patients with mucosa invasion.

The median survival of all the patients with SCLC was 161 (IQR, 46–348) days; 206 (IQR, 79–403) days for limited stage patients, and 137(IQR, 40–314) days for extensive stage patients (Table 2). Using Kaplan-Meier analysis, survival time was measured from the date of diagnosis, and death from all causes during the period of follow up was taken as the outcome. Patients with mucosal invasion had worse survival than patients without (Hazard Ratio [HR] 2.03, 95% CI 1.55–2.42, p<0.0001) (Figure 2). The median survival for patients with endobronchial mucosa invasion was 145 (IQR, 39–322) days, while the median survival for those without mucosal invasion was 290 (IQR, 129–626) days. In extensive stage patients, patients with endobronchial mucosa invasion had worse survival than those without (HR 1.8, 95% CI 1.27–2.33, p = 0.0005) (Figure 3) with a shorter median survival of 125 days (IQR, 31–277 days, P<0.001) compared with that of patients with intact mucosa (207 days, IQR, 123–449 days). In limited stage patients, patients with endobronchial mucosa invasion also had worse survival than those without (HR 2.27, 95% CI 1.57–3.04, p<0.0001) (Figure 4) with a shorter median survival of 180 days (IQR, 60–353 days; P<0.001) compared with that of patients with intact mucosa (460 days, IQR, 215–742 days).

**Table 2:** Survival days of patients.

	Median Survival, days	(IQR^a^)	*P-*value
**Whole cohort**	161	46–348	
**Clinical staging**			
Limited	206	79–403	.001
Extensive	137	40–314	
**Mucosa involvement**			
Involved	145	39–322	<.0001
Uninvolved	290	129–626	
**Limited stage plus**			
Mucosa involved	180	60–353	<.0001
Mucosa uninvolved	460	215–742	
**Extensive stage plus**			
Mucosa involved	125	31–277	<.0001
Mucosa uninvolved	207	123–449	

a IQR: Inter-quartile range.

### Univariate Analysis

Univariate analysis (Table 3) allowed us to identify the possible factors potentially associated with survival time less than the median survival (161 days) in SCLC patients of the whole cohort. We analyzed the association between decreased survival days and variables including age, gender, performance status, clinical staging, mucosal invasion, and administration of therapy of the patients. The results revealed that age (OR, 1.04; 95% CI, 1.02–1.05), extensive stage (OR, 1.61; 95% CI, 1.10–2.36), and endobronchial mucosal invasion (OR, 2.21; 95% CI, 1.28–3.80) were significantly associated with a decrease in survival, while good performance status with ECOG scale ≤2 (OR, 0.19; 95% CI, 0.12–0.30) and receiving chemotherapy more than 4 cycles (OR, 0.41; 95% CI, 0.30–0.56) are associated with a better survival. In patients with measurable tumor size, univariate analysis revealed no difference in size between patients with longer survival and those with shorter survival (5.70±3.74 vs 5.61±2.22, p = 0.432; respectively).

**Table 3:** Univariate analysis of factors potentially associated with survival time that is less than the median survival in SCLC^a^ patients.

	Survival <161days N = 216	Survival ≥161days N = 216	Odds ratio	95% Confidence interval	*P-*value
Age, yr, mean±SD^b^	69.4±9.6	63.4±10.2	1.04	1.02–1.05	<.0001
Sex (Female), no (%)	13(6.0%)	20(9.3%)	0.63	0.30–1.30	.277
ECOG^c^ scale ≤ 2	113(52.3%)	184(85.2%)	0.19	0.12–0.30	<.0001
Extensive stage	135(62.5%)	110(50.9%)	1.61	1.10–2.36	.020
Tumor Size, cm, mean±SD^b^	5.70±3.74	5.61±2.22	0.58	0.21–1.58	.432
Mucosal involvement	193(80.4%)	171(79.2%)	2.21	1.28–3.80	.005
Best supportive care	75(34.7%)	59(27.3%)	1.18	0.94–2.13	.112
Radiotherapy	5(2.3%)	7(3.2%)	0.83	0.42–1.80	.771
CCRT^d^	29(13.4%)	18(8.3%)	0.59	0.31–1.09	.122
Chemotherapy	122(56.5%)	192(88.9%)	0.41	0.30–0.56	<.0001

a SCLC: Small cell lung cancer.

b SD: Standard deviation.

c ECOG: The Eastern Cooperative Oncology Group performance status.

d CCRT: Concurrent chemo-radiotherapy.

### The Cox’s Proportional Hazard Analysis

The Chi-squared test revealed high correlations between good performance status with ECOG scale ≤2 and chemotherapy received (p<0.0001). However, there was no significant correlation identified among age, extensive stage, endobronchial mucosal invasion, and chemotherapy received. Therefore, age, extensive stage, endobronchial mucosal invasion, and chemotherapy received were introduced into Cox’s proportional hazard model. The results (Table 4) showed that age (HR, 1.04; 95% CI, 1.03–1.06), extensive stage (HR, 1.39; 95% CI, 1.06–1.84), and endobronchial mucosal invasion (HR, 2.01; 95% CI, 1.30–3.10) were independent contributing factors for shorter survival, while chemotherapy received (HR, 0.32; 95% CI, 0.25–0.42) was an independent contributing factor for better outcome.

**Table 4:** Cox proportional hazard ratio analysis of SCLC^a^ patients with a survival time less than the cohort median survival.

	Hazard ratio	95% confidence interval	*P*-value
Age	1.04	1.03–1.06	<.0001
Extensive stage	1.39	1.06–1.84	.019
Mucosa involvement	2.01	1.30–3.10	.002
Chemotherapy	0.32	0.25–0.42	<.0001

a SCLC: Small cell lung cancer.

## Discussion

This study demonstrated that 84% (364/432) of SCLC patients had endobronchial mucosal invasion by cancer cells while the cancer was initially diagnosed. Although the baseline characteristics were similar between patients with and without endobronchial involvement, SCLC patients with endobronchial mucosal involvement were at significantly higher risk of decreased survival times even after adjusting subjects’ age, sex, clinical staging, and administrated therapy. Among SCLC patients of either limited or extensive stages, the median survival for patients with endobronchial mucosal invasion was shorter than those without, respectively.

Many studies had tried to identify the clinical and laboratory parameters to predict long-term survival in small cell lung cancer [6], [7], [13], [14]. Compatible with previous reports, our results also indicated that age [6] and extensive stage [7] were independent contributing factors for shorter survival time, while receiving chemotherapy [7] no less than 4 courses was an independent contributing factor for better outcome. Recently, other prognostic factors such as both baseline circulating tumor cell number and change in circulating tumor cell number after chemotherapy have been reported as an independent prognostic factor for SCLC [15]. A previous study reported a much higher rate of relapsing tumor and a worse response to chemotherapy in SCLC patients with abnormal findings on bronchoscopic examination [11]. However, their study did not employ multivariate analysis to evaluate the independent predictive power of endobronchial mucosal involvement on survival. By means of Cox proportional hazard model, our study discloses that endobronchial mucosal invasion is an independent prognostic factor for poor outcome in SCLC patients even under controlling of other associated variables. The predictive power for poor survival of endobronchial mucosal involvement is as strong as that of extensive stage. Therefore, apart from currently recognized prognosticators, circulating tumor cell number and endobronchial mucosal invasion may be taken into consideration for risk stratification of patients in clinical practice and helping clinicians make appropriate treatment decisions for individual patients.

Although it is believed that SCLC arises from or is related to the pulmonary neuroendocrine cells [2], [3], emerging evidence also indicates the possibility that a non-neuroendocrine cell, such as adult lung stem cells and progenitor cells [16], can adopt neuroendocrine characteristics through gene mutation [17]. The key step of endobronchial mucosa invasion is disruption of basement membrane by cancer cells. Previous studies [18], [19] have proposed a model of invasion and metastasis by cancer cells in which the basement membrane (BM) plays an important role as a barrier against tumor cell invasion [20]. There have been several reports claiming that disruption of the BM in various tumors significantly correlated with tumor progression and therefore with reduced patient survival [21]–[24]. The association between endobronchial mucosa invasion and poor survival in SCLC patients could be attributed to higher tumor invasiveness with release of proteolytic enzymes resulting in degradation of BM. The expression of E-cadherin, a calcium-dependent cell-cell adhesion receptor that restricts invasion of cells and reduces metastasis, in tumor cells offers a favorable overall survival in SCLC patients [25]. On the other hand, activation or alternation of c-Met pathway leads to higher cell proliferation and invasion capacity is associated with poor prognosis in SCLC [25]–[27]. Although miRNAs were proposed to be implicated in alternation of SCLC tumor biological behavior, such as tumor invasiveness or chemoresistance [28], [29], a recent study did not find any reported miRNA was prognostic or related to malignant behavior in SCLC patients [30]. Disruption in epigenetic control of cell proliferation, including DNA methylation [31], [32] has been shown to contribute to prolonged and uncontrolled cell survival. Further study is warranted to investigate whether there are aberrant miRNA profiles or epigenetic disruption, or expression of E-cadherin or c-Met activation in SCLC with high potential for endobronchial mucosal invasion.

Even stratified as limited or extensive stage, the presence of endobronchial mucosa invasion among SCLC patients also predicts a decreased survival in each subgroup. Interestingly, the median survival time (207 days) for patients with extensive staged SCLC and endobronchial mucosa invasion is similar to the median survival for patients with limited staged SCLC in the whole cohort (206 days). The presence of endobronchial mucosa invasion may be used to identify the patients at higher risk for poor outcome. Thereafter, routine bronchoscopic examination for endobronchial invasion and frequent monitoring during treatment may be considered for all the SCLC patients. For patients with endobronchial mucosa invasion, even in limited stage, intensive therapeutic intervention may be considered. However, the impact of endobronchial invasion on SCLC outcomes needs to be confirmed by further prospective studies. In addition, the combination of Veteran Affair Administration Lung Cancer Study Group staging system and presence of endobronchial mucosa invasion may provide a better predictive power for prognosis in patients with SCLC.

In conclusion, the present study showed that SCLC patients with endobronchial mucosa involvement had shorter survival time than patients without endobronchial mucosa involvement. The endobronchial mucosa involvement is an independent predictor for decreased survival in SCLC patients, even after taking age, sex, clinical staging, and administrated treatment into account. Our study advances the notion that detection of mucosa invasion by bronchoscopic examination is mandatory in patients with SCLC. The presence of endobronchial mucosa involvement may offer a new predictor of outcome and be used in risk stratification among SCLC patients.

## Supporting Information

Document S1 **An IRB certificate of approval of the study in English version.**
(PDF)

Document S2 **Representative patient’s signed permit for bronchoscopic examination in English Version.**
(PDF)

Document S3 **Representative patient’s signed permit for bronchoscopic examination in Chinese Version.**
(PDF)
