# Supplementary material for: Endobronchial Mucosa Invasion Predicts Survival in Patients with Small Cell Lung Cancer
Source: PLoS One. 2012 Oct 4;7(10):e47613. doi: 10.1371/journal.pone.0047613 (PMC3464219; doi:10.1371/journal.pone.0047613)
Supplement: Document S1 — An IRB certificate of approval of the study in English version. (PDF) [file pone.0047613.s001.pdf]

Chang Gung Medical Foundation
199, TUNG HWA NORTH ROAD,
TAIPEI, TAIWAN, 10507
REPUBLIC OF CHINA
TEL: (03) 3196200
Fax: (03) 3196102

2012-7-11

Protocol Title: The Clinical Value of Bronchoscopy in Predicting Survivals in Patients with Small Cell Lung Cancer

CGMF IRB No.: 99-2011B
Principal Investigator: Chun-Yu Lo
Co-Investigator: Pai-Chien Chou
Protocol Version: 2010.7.16

, was approved by the Institutional Review Board (the "IRB") of Chang Gung Medical Foundation on 2010-9-17. The IRB is organized and operates according to Good Clinical Practice and the applicable laws and regulations.

Sincerely Yours,

T. T. Hsieh

Tsang-Tang Hsieh, M.D.
Chairman
Institutional Review Board
Chang Gung Medical Foundation
