# Supplementary material for: Endobronchial Mucosa Invasion Predicts Survival in Patients with Small Cell Lung Cancer
Source: PLoS One. 2012 Oct 4;7(10):e47613. doi: 10.1371/journal.pone.0047613 (PMC3464219; doi:10.1371/journal.pone.0047613)
Supplement: Document S2 — Representative patient’s signed permit for bronchoscopic examination in English Version. (PDF) [file pone.0047613.s002.pdf]

LLEBR120000003

Examination/Minor Procedure Consent Form (Representative)

Patient Medical Record No: ______ Passport No. : ____________________

Patient, ____________________ (name of patient), sex: ☑ male ☐ female, born on ____________ (Y/M/D), diagnosed with lung mass

, hereby consent to undergo bronchoscopic examination, including bronchosopic biopsy (name of examination/minor procedure)

The nature, purpose, and necessity for the examination/procedure, the success rate, the possible complications, risks and benefits of the examination/procedure have been explained to signer by Dr. ____________ (name of medical practitioner) in ____________ (language) with the assistance of ____________ (name of interpreter)

I/We also consent to further measures as may be deemed necessary during the course of the examination/procedure. The use of drugs and medicines as may be deemed advisable or necessary for the said examination/procedure.

Consent given by

Signature of the consenting parties ____________ Relationship to the patient ____________

Telephone ( ) ____________ Date ____________ (Y/M/D) Time ____________

Address ____________________________________

Witnessed by

Name of witness ____________ Relationship to the patient ____________

Telephone ( ) ____________ Date ____________ (Y/M/D) Time ____________

AddressiG ____________________________________

Interpreted by

Name of interpreter ____________ Relationship to the patient ____________

Telephone ( ) ____________ Date ____________ (Y/M/D) Time ____________

Language ____________ Address ____________

Note:

1. The consent form must be signed by the patient personally; however, if the patient is a minor or unable to sign personally, this can be performed by another individual as determined by the Medical Care Act Article 64 Paragraph 2 (Civil LawiG20 years old is the legal age of an adult).
2. Consenting parties who are not the patients scheduled for this examination/procedure, should fill in their relationship to the patient next to "Relationship to Patient".
3. In accordance to the Medical Care Act Article 64 Paragraph 1, if there is a need for a repeat the examination/procedure, after completion of the first examination/procedure, an explanation must be provided and consent must be appropriately taken, except in emergency situations.
4. Medical Care Act Article 64iG"Medical care institutions shall explain the invasive examination or treatment regulated by the central competent authority to the patient or his/her legal agent, spouse, kin, or interested party, and must obtain his/her consent and signature on the letter of consent before commencing with the procedure. However, in case of emergency, the provisions above shall not apply.
   The legal agent, spouse, kin, or interested party may sign the letter of consent referred to in the preceding Paragraph in the case that the patient is a minor or unable to affix the signature personally."
5. The consenting parties should clear all doubts or seek additional explanations from the doctor before signing the consent.

The copy has been delivered to :

DuplicateiG(1)physiciani÷Patienti÷Medical Records Room
