# Supplementary material for: Endobronchial Mucosa Invasion Predicts Survival in Patients with Small Cell Lung Cancer
Source: PLoS One. 2012 Oct 4;7(10):e47613. doi: 10.1371/journal.pone.0047613 (PMC3464219; doi:10.1371/journal.pone.0047613)
Supplement: Document S3 — Representative patient’s signed permit for bronchoscopic examination in Chinese Version. (PDF) [file pone.0047613.s003.pdf]

胸腔內科檢查同意書

一式一聯：醫師→病人→存病歷

病人＿＿＿＿＿＿＿（病歷號碼＿＿＿＿＿），性別＿＿，＿＿＿＿＿生，因＿＿＿＿＿＿＿＿＿＿＿＿＿＿＿＿＿＿＿＿

，有接受胸腔內科檢查＿＿＿＿＿＿＿＿＿＿＿＿

之必要，立同意書人經　貴院＿＿＿＿＿＿＿＿醫師（由醫師親自簽名）

（開立醫囑之醫師及施行診療之醫師如有不同，均應向病人說明後於本欄位簽名。）

詳細說明，已充份瞭解下列事項：

（一）施行檢查之原因及其必要性。

（二）檢查可能發生之併發症及危險。（詳如背頁說明）

（三）病人提出之問題與醫師回覆說明如下：

＿＿＿＿＿＿＿＿＿＿＿＿＿＿＿＿＿＿＿＿＿＿＿＿

＿＿＿＿＿＿＿＿＿＿＿＿＿＿＿＿＿＿＿＿＿＿＿＿

茲同意病人接受該項檢查，貴院應善盡醫療上必要之注意，若發生緊急情況時並同意接受貴院逕行為必要之處理。

此致

長庚醫療財團法人

立同意書人：　　　　　　　　簽章

身分證編號：

住址：

電話：（　　）

關係：病人之

中　華　民　國　　　　年　　　　月　　　　日

附註：一、立同意書人需由病人親自簽具；但病人如為未成年人或不能親自簽具者，得由醫療法第六十四條第二項規定之人員簽具。(民法規定：年滿20歲為成年人)

二、立同意書人非病人本人者，應填載與病人之關係。

三、醫院為病人實施侵入性檢查或治療後，如有再度實施之必要，除有醫療法第六十四條第一項但書所訂情況緊急者外，仍應再度說明，並另簽具同意書，始得為之。

四、醫療法第六十四條規定：「醫療機構實施中央主管機關規定之侵入性檢查或治療，應向病人或其法定代理人、配偶、親屬或關係人說明，並經其同意，簽具同意書後，始得為之。但情況緊急者，不在此限。
前項同意書之簽具，病人為未成年人或無法親自簽具者，得由其法定代理人、配偶、親屬或關係人簽具。」

五、立同意書人請務必詳閱背後說明，如有任何疑問或須更進一步瞭解，請於簽具本同意書前，詳細詢問相關醫師。

胸腔內科檢查說明

一、支氣管鏡檢查及其後續檢查：

檢查中可能發生喉頭攣縮、呼吸窘迫、低血壓昏厥、暫時性血壓降低等併發症，亦可能發生呼吸道出血、心律不整等現象，若加做生檢切片、擦刷抹片、經氣管肺切片、經氣管細針抽吸抹片、雷射燒灼、異物取出等，則併發症發生機率增高，且亦可能發生氣胸、血胸、咳血等併發症，一般此類併發症多屬輕度稍加治療即可恢復，但偶而也有發生嚴重併發症甚至危及生命之可能。一般而言，嚴重併發症及死亡概率為萬分之一。檢查後少部份病人會發生短暫發燒、血痰、肺部感染等。

二、螢光支氣管鏡檢查：

透過螢光之特殊波長及正常、不正常之螢光激發測試，確定原位癌細胞病變位置，以利準確取得細胞進行DNA分析，作為治療前後之評估，並可鑑別診斷早期病變，達到早期預防及治療之功能，提高存活率。

三、超音波及其相關檢查：

超音波檢查本身應不具危險性，但其相關檢查如肋膜腔積液抽取、肋膜切片、淋巴腺或腫瘤針吸抽取、經皮穿胸細針活體生檢或胸管放置等，則有發生併發症之可能。一般之併發症為氣胸、血胸、皮下氣腫感染等，輕微併發症可能不需治療，中度者稍加治療即可，極少數會發生致死併發症。

立同意書人簽名：
